# Characteristics of Lightweight Foam Concrete Manufactured Using Water-Soluble Polymers and Lightweight Aggregates

**DOI:** 10.3390/ma18081881

**Published:** 2025-04-21

**Authors:** Choonghyun Kang, Ki-Young Seo, Yong-Myung Park, Taewan Kim

**Affiliations:** 1 Department of Ocean Civil Engineering, Gyeongsang National University, Tongyeong 53064, Republic of Korea; 2 HK E&C, 31, Beomeocheon-ro, Geumjeong-gu, Busan 46220, Republic of Korea; 3 Department of Civil Engineering, Pusan National University, Busan 46241, Republic of Korea

**Keywords:** polyvinyl alcohol, perlite, cenosphere, ultralight foam concrete

## Abstract

This study aimed to analyze the effects of PVA aqueous solution as a new foaming agent, and the production and characteristics of ultralight foam concrete using a mixed lightweight aggregate of perlite (PL) and cenosphere (CP). In addition, the application of a new high-temperature curing process was proposed to improve the foaming effect of PVA and reduce the weight of concrete. The mixing ratios (s/c) of the PVA solution and OPC were 1.0, 1.5, and 2.0, and the ratio of the PVA solution–OPC–lightweight aggregate (perlite and cenosphere) (s/(c + CP + PL)) was 0.43–1.0. As a result, an ultralight foam concrete with a dry density of less than 1.0 g/cm^3^, an average pore diameter of 0.1–2.3 mm, and a compressive strength of 1.5–10.5 MPa could be manufactured. From the experimental results, PVA showed sufficient usability as a foaming agent. And the new high-temperature curing process proposed in this study could be suggested as a method applicable to the expansion of pores and lightweight reduction in the manufacture of foamed concrete. The diameter of the foamed pores changed depending on the mixing ratio of CP and PL, and the diameter of the foamed pores increased as the ratio of PL increased. However, an increase in the ratio of CP improved the insulation properties. The increase in the OPC ratio increased the mechanical strength, but increased the dry density and decreased the insulation properties. Therefore, the mixing ratio of CP and PL was an important factor affecting the properties of ultralight foam concrete. From the experimental results, PVA was suggested to have sufficient potential as a new foaming agent, and the new high-temperature curing process proposed in this study is expected to be applicable to the production of foam concrete using PVA.

## 1. Introduction

Polyvinyl alcohol (PVA) is a water-soluble polymer and is used in various fields as a safe material with very little impact on living organisms and the human body [1,2,3]. Recently, it has been widely used in the food and medical fields [2,3,4]. In particular, PVA is manufactured in the form of fiber and used together with concrete, and is widely used as fiber-reinforced concrete. PVA fiber-reinforced concrete is used to manufacture high-performance concrete due to its improved ductility and toughness [5,6,7]. However, there are not many studies using PVA mixed in concrete compared to PVA fiber reinforced concrete [8,9,10,11,12]. The properties of concrete using PVA are gradually being studied through various experiments.

Research on concrete or cement using PVA published to date is mainly about the aggregate surface modification, mechanical properties and durability of PVA-modified cement/concrete. Kou and Poon [13] evaluated the effect of impregnation of recycled concrete aggregate (RCA) with polyvinyl alcohol (PVA) at concentrations of 6%, 8%, 10%, and 12% on the strength and durability properties of concrete. The results showed that mechanical properties were improved, shrinkage was reduced, water absorption was reduced, and chloride ion penetration resistance was increased. Huiwen et al. [14] showed that the mechanical performance of recycled aggregate pretreated with PVA polymer solution and a lyophilized activator was improved because the porosity was reduced, the width of the interfacial transition zone (ITZ) was effectively narrowed, and the ITZ structure became much denser. Mannan et al. [15] attempted to improve the quality of oil palm shell (OPS) to be used as coarse aggregate by applying various pretreatment methods. They reported that the pretreatment method using the PVA solution showed the best performance in improving the quality of OPS aggregate and OPS concrete. Thong et al. [16] reported that PVA improved the adhesion between paste and aggregate by decreasing the ITZ thickness and Ca(OH)_2_ formation between aggregate and paste. Allahverdi et al. [17] investigated the effects of the water–cement (W/C) ratio and polymer–cement (P/C) ratio simultaneously on the flexural strength of V-type portland cement paste. They reported that there was an optimum value for both the W/C ratio and the P/C ratio, which resulted in increased strength, increased dry bulk specific gravity, and significantly reduced total permeable pore volume and water absorption of the specimens. Ohama [18] reported the overall characteristics and effects of polymer modification using water-soluble polymers and liquid polymers on cement mortar and concrete. He reported on the improvement of properties and areas for improvement of various polymer-modified concrete. Kim et al. [19] investigated the structure and properties of mortar and concrete containing 1 wt% and 2 wt% polyvinyl alcohol (PVA) based on cement weight. Experimental results showed that adding PVA increased the void ratio and apparent fluidity, and decreased the bleeding of fresh mortar and concrete. Kim and Roberetson [20] confirmed that adding a large amount of polyvinyl alcohol (PVA) to cement paste was effective in increasing the aggregate-paste bond strength. They reported that the increase in bond strength was due to the suppression of the porous interfacial transition zone and the inhibition of calcium hydroxide nucleation on the aggregate surface. Wu et al. [21] evaluated the freeze–thaw resistance ability of concrete added with cellulose/PVA solution. They reported that the addition of cellulose/PVA hydrogel maintained the compressive strength after freeze–thaw cycles, promoted the initial hydration of cement, and formed a multi-scale pore structure to reduce the osmotic pressure and swelling pressure. Morlat et al. [22] observed that the fracture stress and the stored energy at fracture of the specimens improved as the molecular weight of PVA increased. They confirmed that the dissipated energy measured using crack opening displacement CMOD increased in polymer modified cement paste using PVA aqueous solution. Pique and Vazquez [23] found that the flexural strength increased and the compressive strength decreased in the portland cement paste modified with PVA. They found that the addition of three different montmorillonites (MMTs) to the cement paste containing PVA had little effect on the mechanical properties. Liu et al. [24] studied the effect of PVA on the rheological properties of cement mortar according to the degree of hydrolysis and polymerization. The rheological properties of PVA modified cement mortar could be explained by the modified Bingham model, and it was reported that the yield stress and plastic viscosity of the modified cement mortar increased rapidly with increasing polyvinyl alcohol content and degree of polymerization. Kim and Robertson [25] conducted a study to remove the air bubbles formed when cement and aggregates are mixed with PVA, an aqueous polymer solution. They proposed a method of pre-wetting cement and sand with water, and then adding a concentrated PVA solution. As a result, they confirmed that the air content of the PVA-modified mortar was reduced.

This is greatly affected by the type and concentration of PVA. And many previous studies have reported a decrease in mechanical performance due to the use of PVA, and the main causes are cited as air bubbles generated by mixing PVA and PVA reducing the hydration reaction of cement particles. And many previous studies have reported a decrease in mechanical performance due to the use of PVA, and the main causes are cited as air bubbles generated by mixing PVA and PVA reducing the hydration reaction of cement particles. This has limited the use of PVA in concrete or cement. As a result, it is difficult to present a clear trend or manufacturing process because the use of PVA in concrete is affected by various factors [17,19].

Expanded perlite is a chemically inert, amorphous aluminosilicate volcanic glass with a very small volume density of 80 kg/m^3^ to 240 kg/m^3^ and dense micropores [26,27,28]. In the construction field, perlite is utilized for its lightweight or thermal conductivity reduction properties. Therefore, perlite is widely used in the development of lightweight structures [29,30] or insulation materials [30,31,32], and many research results have been reported. However, perlite substitution is mentioned as a problem that needs to be improved due to the decrease in mechanical performance [26,28,33] compared to its high lightweight and insulation performance. The cenosphere (CP) is an industrial byproduct with chemical properties similar to fly ash, consisting of silicon dioxide and aluminum oxide [34,35]. CP is mainly used in the manufacture of lightweight concrete due to its hollow microspheres and various sizes and low densities [34,36]. It is also used in the manufacture of concrete products that improve both lightweight and insulation properties [37,38,39]. Research using CP is mainly focused on the manufacture of concrete that focuses on (ultra) lightweight or insulation properties, and experiments on mechanical performance and durability are also being conducted [40,41,42]. However, like PL, CP also has the problem that its mechanical performance decreases as the substitution ratio increases [38,42].

CP and PL are inorganic materials mainly used for the manufacture of lightweight concrete and the purpose of improving insulation properties. This is because many previous studies have confirmed the superiority of lightweight and insulation performance of concrete containing CP and PL. Therefore, in this study, two types of lightweight materials, CP and PL, were mixed in various ratios to obtain ultra-lightweight characteristics and the effect on the foaming characteristics by PVA was investigated.

This study focuses on the formation of micropores, which is one of the several disadvantages of concrete using PVA. Micropores generated by PVA are one of the main factors that reduce the density of the matrix and reduce the mechanical performance. However, if the effect of micropores can be maximized and the foaming agent can be used in the production of foamed concrete, it is believed that PVA foamed concrete utilizing the various advantages of PVA mentioned above can be manufactured. To this end, the possibility of manufacturing lightweight foamed concrete using lightweight aggregates in OPC-based concrete was examined. Two types of lightweight aggregates were used: perlite (PL) and cenosphere (CP). In order to secure lightweightness, the amount of OPC was minimized, and the mechanical properties of lightweight foamed concrete according to the ratio of PL and CP were analyzed to have ultra-lightness of less than 1.0 g·cm^3^ in dry density. It is expected that ultralight concrete can be applied to the development of floating concrete and high-performance insulation materials through additional analysis and experiments.

In order to maximize the foaming effect of PVA, a new curing process was introduced in this experiment. A new curing method is a high-temperature curing method. High-temperature wet curing (60 °C, RH ≥ 90%) was applied for 12 h to promote the initial hydration reaction of OPC and the expansion of the pores formed by PVA, and then high-temperature dry curing (105 ± 5 °C, RH ≤ 30%) was applied for 12 h to remove moisture from PVA and promote hardening. The samples that went through the high-temperature curing process for 24 h were cured at the general room temperature in the laboratory (23 ± 5 °C, 40% ≤ RH ≤ 60%). The new high-temperature curing method is expected to improve the initial strength by expanding the pores formed by PVA and promoting the hydration reaction of OPC.

The ultimate objectives of this study are threefold: (i) to examine the effectiveness and potential of PVA as a new foaming agent, (ii) to apply a new high-temperature curing process to enhance the foaming effect, and (iii) to verify the production of ultra-lightweight concrete and its mechanical performance according to the foaming effect of PVA and lightweight aggregates mixed with CP and PL. It is expected that the results of this study will provide important basic and reference data for the development of ultra-lightweight foamed concrete through various applications and performance enhancement in follow-up studies.

## 2. Materials and Methods

### 2.1. Materials

**Figure 1:**
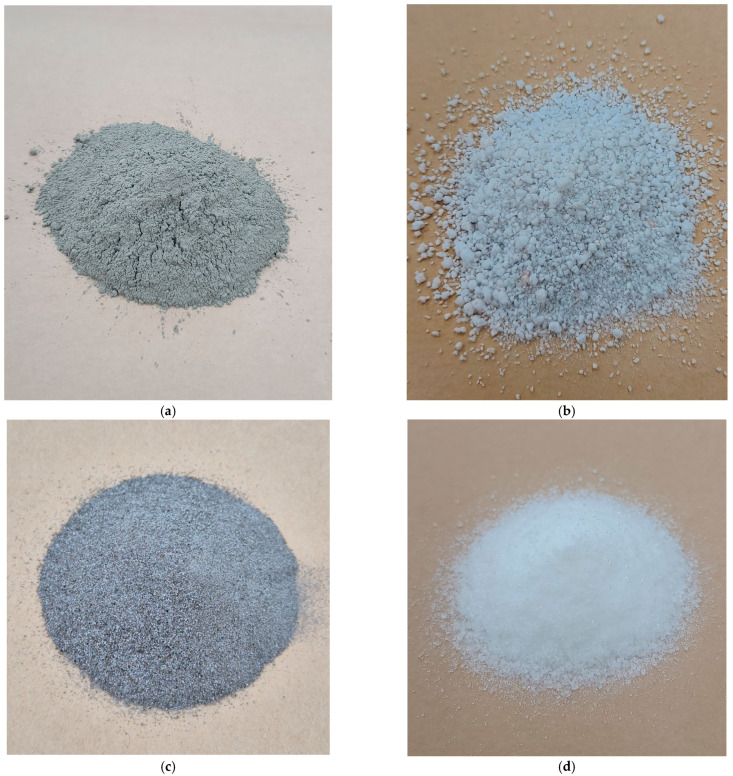
Photos of raw materials: (**a**) OPC, (**b**) PL, (**c**) CP, and (**d**) PVA.

The components of ordinary Portland cement (OPC), cenosphere (CP) and perlite (PL) used in the experiment were analyzed using X-ray fluorescence (XRF), and the results are shown in Table 1, respectively. The OPC was supplied by Hanil Cement (Busan, Republic of Korea), CP was supplied by Seo Kyung CMT (Busan, Republic of Korea), and PL was supplied by Kyungdongwon (Paju-si, Republic of Korea). The density of PL is 0.11 g/cm^3^, thermal conductivity is 0.03–0.05 W/mK, the particle size is 10–100 μm, and the average diameter is 50 μm. The particle diameters of CP are 10–350 µm, the average diameter of 180 µm, the bulk density is 0.42 g/cm^3^, and the specific gravity is 0.65 g/cm^3^. PVA is a product of Kuraray (Tokyo, Japan), and is a white powder type product with viscosity of 20.5–24.5 mPa·s, hydrolysis of 87.0–89.0 mol%, molecular weight of 83,000, and pH of 7.0. The Figure 1 shows OPC, CP, PL, and PVA. The CP requires approximately three times higher cost per unit-g compared to PL. This is believed to be because, compared to PL, which is a natural material, CP requires special capture equipment during the combustion process of coal-fired power plants, capture-transport-storage process, and additional CP selection process [33,34].

### 2.2. Experiments

In this study, the concentration of the PVA solution was selected as 5.0% based on reports of various previous studies using PVA [20,21,23] and preliminary experiments. If it exceeds 5.0%, the viscosity of the PVA solution increases depending on the nature, concentration, temperature, and mixing materials of PVA, and the degree of viscosity increase and the range of change increase, making it difficult to obtain a concrete mix of uniform quality. Therefore, 5.0% was selected considering the foaming effect of the pores by PVA and the viscosity of the PVA solution.

The mixing ratio was considered in two series. The detailed mixing ratio is presented in Table 2. The first series is a mixing ratio in which the ratio of CP and PL is changed in six ways. At this time, the total of the PVA solution, OPC, and lightweight aggregate (CP + PL) is constant. That is, in Table 2, the mass ratio of the PVA solution and OPC, s/c, is constant at 1.5, the weight sum of CP and PL is constant at 100 g, s/(CP + PL) is 3.0, and the c/(CP + PL) ratio is constant at 2.0. In addition, the ratio of the sum of OPC and lightweight aggregate to the weight of the PVA solution, s/(c + CP + PL), is also constant at 1.0. The first series is to compare and analyze the foaming characteristics and mechanical performance according to the change in the ratio of CP and PL.

The second series is based on the experimental results of the first series, and the amount of OPC was changed in two ways for a constant PVA solution so that the s/c ratio was 2.0 and 1.0. This is to compare and analyze the foaming characteristics and mechanical performance of lightweight concrete according to the change in the amount of OPC. In addition, the amount of PL was kept constant for each OPC, and the amount of CP was changed into three types to measure the effect of CP on lightweight and foaming characteristics. Accordingly, the s/(CP + PL) ratio ranges from 0.77 to 1.58, the c/(CP + PL) ratio ranges from 0.38 to 1.58, and the s/(c + CP + PL) ratio ranges from 0.43 to 0.88.

The two series are to compare and analyze the effect of the change in the mixing ratio of OPC-CP-PL on the foaming characteristics and mechanical performance of ultra-lightweight foam concrete and to suggest the optimal mixing ratio required for product manufacturing.

Prepare tap water heated to 90–100 °C, add the specified amount of PVA and stir for 2–5 h using a magnetic stirrer. Add a set amount of PVA to tap water at 90–100 °C in a stirrer with a temperature maintenance device at 90–100 °C and stir for 2–5 h with a magnetic stirrer. The PVA solution in which the PVA particles are completely dissolved is left to stand for about 6 h at room temperature in the laboratory and cooled. After weighing OPC, PL, and CP according to the mixing ratio, add them to the mixing bowl, and then add the PVA solution cooled to room temperature and mix. Mixing was performed using a mixing device suggested in ASTM C305 [43], at a low speed (140 ± 5 r/min) for 60 s, stopped for 30 s, and then at a medium speed (285 ± 10 r/min) for 120 s. During the 30 s stop in the middle of the mixing process, scrape down into the batch any paste that may have collected on the sides of the bowl. After mixing, pour into a 50 mm cubic mold, smooth the surface, and place in a high-temperature and humid curing chamber (60 °C, RH ≥ 90%). After 12 h, take the samples out of the high-temperature and humid curing chamber and place in a high-temperature dry curing chamber (105 ± 5 °C, RH ≤ 30%) for 12 h, then take them out and store them in an environment identical to room temperature in the laboratory (23 ± 5 °C, 40% ≤ RH ≤ 60%).

The distribution, shape, and diameter of pores foamed by PVA were measured by applying an optical method to observe various pores [3]. The measurement method was as follows: Images were taken with an OLYMPUS SZX7 microscope (Olympus Corporation, Tokyo, Japan) and a dedicated camera mounted on the microscope. The pore images of each sample surface were obtained using an optical microscope, and then binarized using Photoshop image processing software. Then, the binarized images were analyzed using Image-J software (version: 1.54m) to obtain the average pore diameter of the macroscopic pores distributed on the sample surface. The compressive strength was measured at 1 and 28 days using the ASTM C109 method [44], and the average of three measurements was used. The samples used to measure the compressive strength were in the shape of a cube with a side length of 50 mm.

The thermal conductivity of the specimens was measured using an MP2 device (Thermtest, Veddige, Sweden). The MP-2 uses a thin coil sensor in the form of a wire, which is heated by a constant current source (q) and records the change in electrical resistance of the wire or the temperature change via a resistance temperature detector. For samples with high thermal conductivity, the resistance increases more slowly over time, while for samples with low thermal conductivity, the resistance increases more quickly over time. In particular, the Transient Plane Source (TPS 4) single-sided (asymmetric) sensor is one of the sensors optimized for the MP-2, and measures low-density and low-thermal-conductivity materials of 0.03 to 5 W/m∙K simply and accurately through the transient plane source (TPS) method. It is mainly used for measuring the thermal conductivity of polymers, ceramics, and composites, and provides an accuracy of 5% and a measurement reproducibility of 2%. The MP2 can be measured up to 0.03–5.0 W/m∙K using a transient planar source sensor (TPS-4) that meets the ASTM D7984 standard [45]. The dimensions of the test specimens used for thermal conductivity measurement were 50 × 50 × 50 mm^3^. Measurement values were obtained from three samples per mixture and the average result was calculated [37]. Dry density of specimens was calculated using the following equation:

$$\rho_{d}=\frac{M_{d}}{V}$$

where $\rho_{d}$ is the dry density, $M_{d}$ is the mass after drying in a dryer at 105 ± 5 °C for 24 h, and *V* is the volume of the test specimen.

## 3. Results and Discussion

### 3.1. Foaming and Microporosity

**Figure 2:**
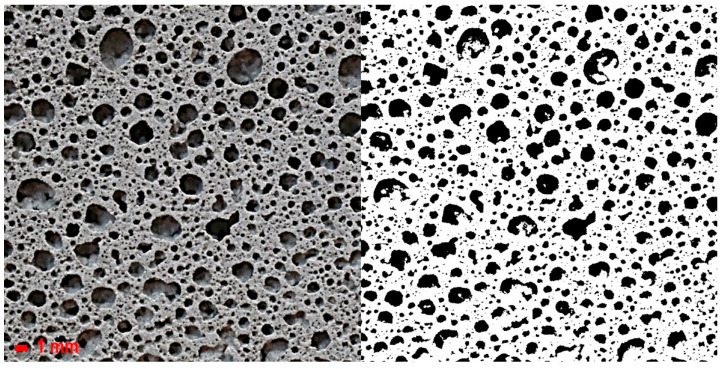
Original images and binarized schematic images.

Figure 2 shows the cross-sectional images of the samples and their binarized images. All samples went through the binarization process to measure the average pore diameter. The pore diameters were measured for three cross-sections in one sample to obtain the average diameter value.

**Figure 3:**
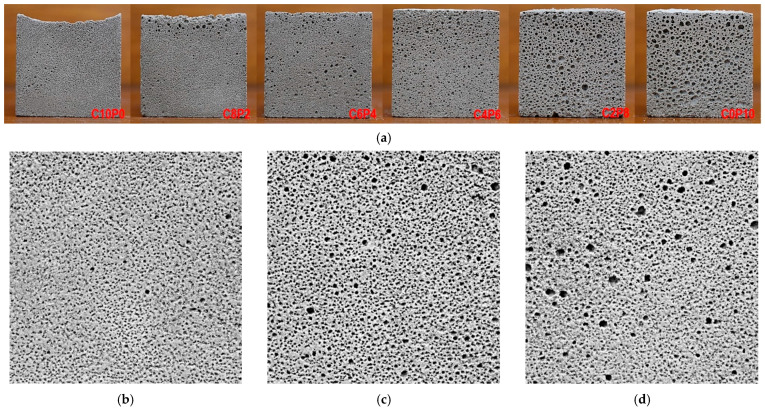

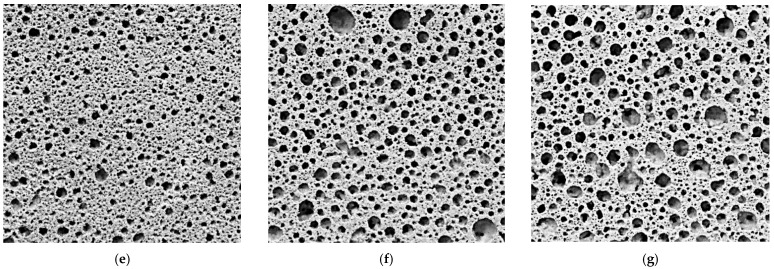
Foam images of the samples: (**a**) the collection of samples, (**b**) C10P0, (**c**) C8P2, (**d**) C6P4, (**e**) C4P6, (**f**) C2P8, and (**g**) C0P10.

Figure 3 shows the foaming characteristics according to the change in the mixing ratio of CP and PL. Figure 3a is a collection of the shapes of all samples for six substitution ratios. The first sample, C10P0, was manufactured with 100% CP, and it can be seen that the upper surface shrank relatively compared to the other samples, resulting in a sagging shape downward. C8P2, with 80% CP + 20% PL, shows much improved sagging of the upper surface due to shrinkage compared to the C10P0 sample. In addition, such sagging of the upper surface of the samples starting from the C6P4 sample, was not observed. It is thought that the occurrence of sagging of the upper surface is because CP hinders the expansion of bubbles by PVA, while PL improves it. As a result, as the substitution ratio of PL increases, shrinkage decreases and the pore diameter gradually expands to reduce shrinkage. This can be thought of as the density of CP (0.42 g/cm^3^) being about 3.8 times heavier than that of PL (0.11 g/cm^3^), which has the effect of inhibiting pore expansion.

The pore expansion inhibition effect due to the density difference between CP and PL can be confirmed as the pore diameter gradually increases as the substitution rate of PL increases. As a result, the pore diameter of the C0P10 sample with 100% PL was observed to be the largest. Therefore, it is judged that the effect on pore expansion is more favorable as the substitution rate of PL increases than that of CP.

**Figure 4:**
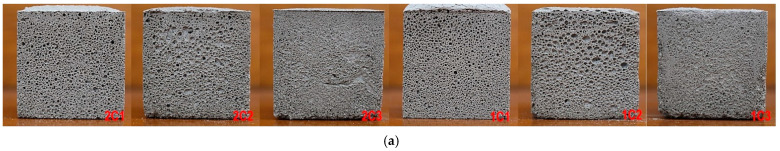

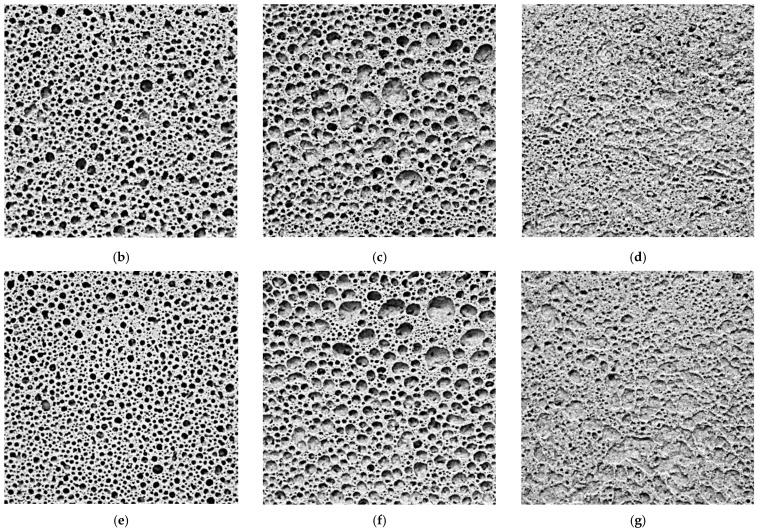
Foam images of the samples: (**a**) the collection of samples, (**b**) 2CP1, (**c**) 2CP2, (**d**) 2CP3, (**e**) 1CP1, (**f**) 1CP2, and (**g**) 1CP3.

Figure 4 shows the samples manufactured to observe the effect of changing the amount of CP for varying the amount of OPC and a constant amount of PL on pore formation and expansion. In Figure 4a, for both samples with s/c ratios of 2.0 and 1.0, the pore diameters were the largest when CP:PL = 2:1 for 2CP2 and 1CP2, and the pores of 2CP3 and 1CP3 for 3:1 were the smallest. That is, when the CP:PL ratio is 1:1 (Figure 4b,e) and 2:1 (Figure 4c,f), there is a pore expansion effect, but when the CP amount is three times that of PL at 3:1 (Figure 4d,g), the pore expansion effect decreases sharply. This is thought to be due to the difference in the densities of PL and CP mentioned above. In addition, the pore diameters expanded more when CP:PL = 2:1 than when it was 1:1, but the size change of the diameters was very large. Figure 4b,e show that most pore diameters are similar and have small deviations. However, in Figure 4c,f, pores of various sizes were observed, from large diameters to very small diameters, and the deviations in diameters were very large. Therefore, it is judged that when the CP:PL ratio is less than 2:1, the diameters of the foamed pores are formed in similar sizes with small deviations.

### 3.2. Average Pore Diameter

**Figure 5:**
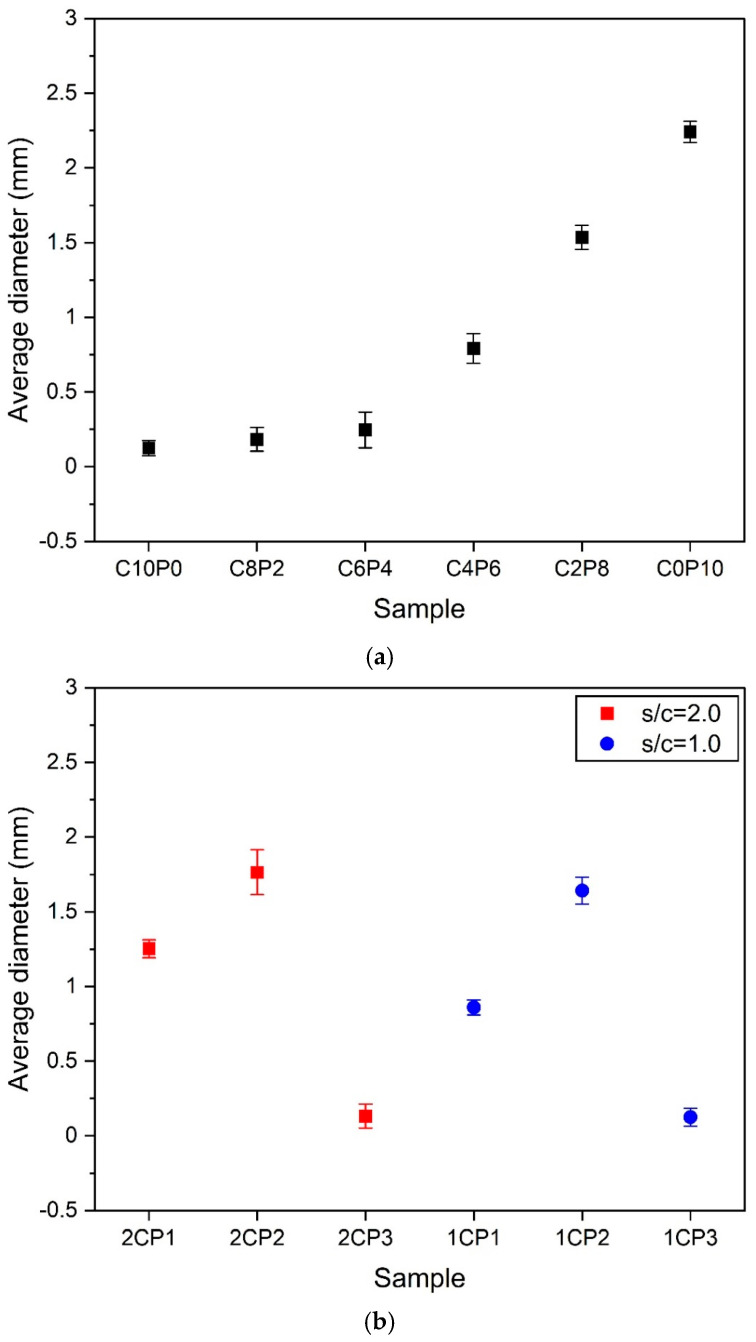
Average pore diameters. (**a**) Samples according to the change in the exchange rate of CP and PL. (**b**) Samples according to the change in the amount of CP when s/c = 2.0 and 1.0.

Figure 5 shows the results of measuring the average diameter of the samples. As mentioned above, the change in the average diameter according to the ratio of CP and PL shown in Figure 3 is shown in Figure 5a. As the substitution ratio of PL increases, the value of the average diameter increases. The average diameter of the 100% CP sample, C10P0, is the smallest, but the pore diameters are similar, so the deviation is very small. However, the 60% CP + 40% PL (C6P4) sample showed the largest deviation value among the six samples. This means that the pore expansion effect due to high-temperature curing was clearly observed from the 40% PL substitution ratio. In other words, it can be considered that the substitution ratio at which the pore expansion inhibition effect decreases and the expansion effect is fully expressed is the substitution ratio at 40% PL substitution ratio. As a result, the variability of the change in the diameter size of the pores increased. After that, the average diameter of the PL substitution samples over 60% gradually increased, while the deviation also decreased. Therefore, when mixing CP and PL as lightweight aggregates, it is thought that the substitution rate of PL has a greater effect on increasing the pore diameter size than that of CP.

Figure 5b shows the effect of CP change on pore diameter change when the s/c ratio is 2.0 and 1.0. The 2CP1 and 1CP1, where the CP:PL ratio is 1:1, showed the smallest pore diameter size variation. However, when it is 2:1 (2CP2, 1CP2), the samples showed a larger average pore diameter than when it is 1:1, but the deviation of the values increased. This is clear from the pore size variation in Figure 4 described in the previous section. 2CP2 and 1CP2 have a mixture of large and small pores. Therefore, the average pore diameter increased, but the pore diameter variation was the largest. In contrast, the 2CP3 and 1CP3 samples, where CP:PL = 3:1, showed a rapid decrease in pore diameter due to the suppression of pore expansion by the increased amount of CP. As a result, the average pore diameter also showed the smallest value. As the amount of OPC increased, the s/c ratio increased from 2.0 to 1.0, reducing the pore expansion effect, and the average pore diameter decreased at all CP:PL ratios. That is, at the same CP:PL ratio, the average diameters of samples with a lower amount of OPC were larger in 2CP1 > 1CP1, 2CP2 > 1CP2, and 2CP3 > 1CP3. This is thought to be because the setting and hardening of the hydration reaction material by the hydration reaction of OPC reduces the pore expansion effect.

The average pore diameter measurement results in Figure 5 show that when mixing two types of lightweight aggregates, CP and PL, the higher the substitution ratio of CP, the more the pore expansion effect is suppressed, and the average pore diameter decreases. In addition, an increase in the amount of OPC also had the effect of suppressing pore expansion. Therefore, it is judged that a mixing ratio that minimizes the mixing ratio of OPC and CP will be effective in expanding pores and enlarging the average pore diameter.

### 3.3. Dry Density

**Figure 6:**
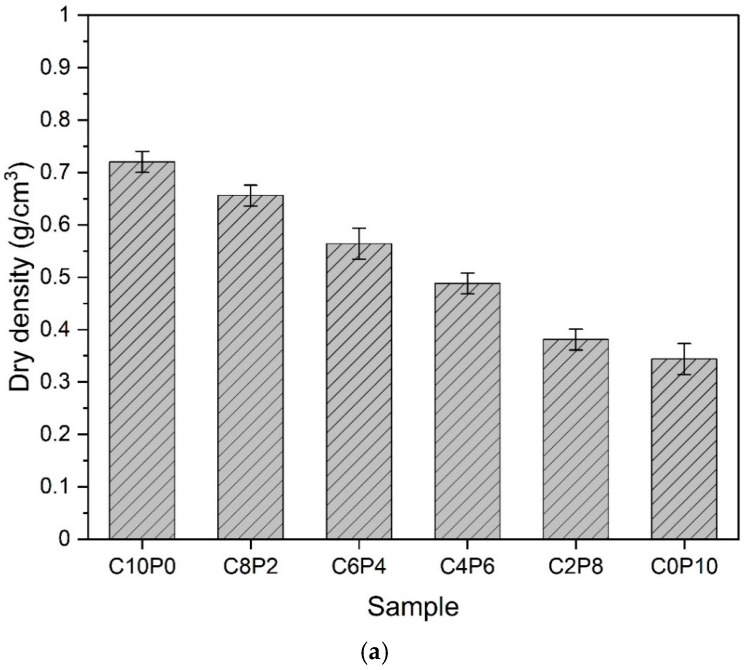

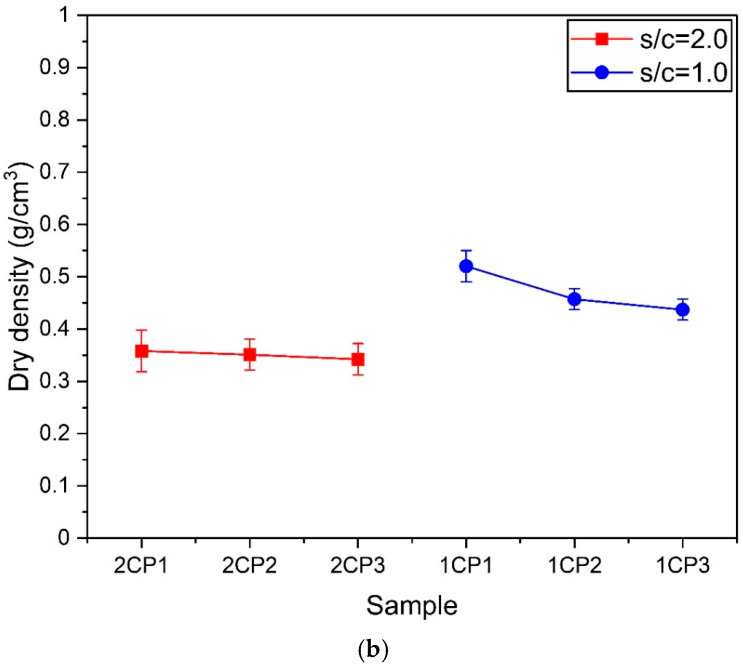
Dry density. (**a**) Samples according to the change in the exchange rate of CP and PL. (**b**) Samples according to the change in the amount of CP when s/c = 2.0 and 1.0.

Figure 6 shows the results of measuring the dry density of the samples. All samples showed dry density values less than 1.0 g/cm^3^, indicating the possibility of manufacturing ultralight foam concrete. In Figure 6a, as the PL replacement ratio increased, the dry density decreased, and the dry density of the C0P10 sample with 100% PL was the lowest. As mentioned in the previous sections, the increase in the CP replacement ratio has the effect of suppressing the expansion of pores, so that the pores hardly expand and the pore diameters are very small. This further enhances the lightness by forming spaces inside the matrix due to the large pore diameters of the samples with a higher amount of PL, thereby reducing the dry density. CP (0.42 g/cm^3^) is about 3.8 times heavier than PL (0.11 g/cm^3^), so the increase in the amount of CP is considered to be a factor that suppresses the expansion of pores.

Figure 6b shows the change in dry density according to the amount of CP when the s/c ratio is 2.0 and 1.0. When s/c = 2.0, the dry density decreases as the amount of CP increases. This is because as the amount of CP increases, the expansion effect of the pores is suppressed, so the volume occupied by the large pores inside the matrix decreases, but the amount of micro pores increases relatively. This is because as the pores expand, the fine micro pores should merge with each other to grow into large pores, but the increase in CP hinders the expansion of these pores. As a result, it is thought that there are many micro pores that do not form large pores because the expansion effect is suppressed. In the samples where s/c = 1.0, the dry density decreased as the amount of CP increased, which is a similar trend to when 2.0 was used. The dry density decreased as the amount of OPC increased. This means that the dry density of the s/c = 1.0 samples was greater than that of the s/c = 2.0 samples. OPC has the heaviest density of 3.15 g/cm^3^, so increasing the amount of OPC results in a decrease in lightness, thus increasing the dry density.

### 3.4. Compressive Strength

**Figure 7:**
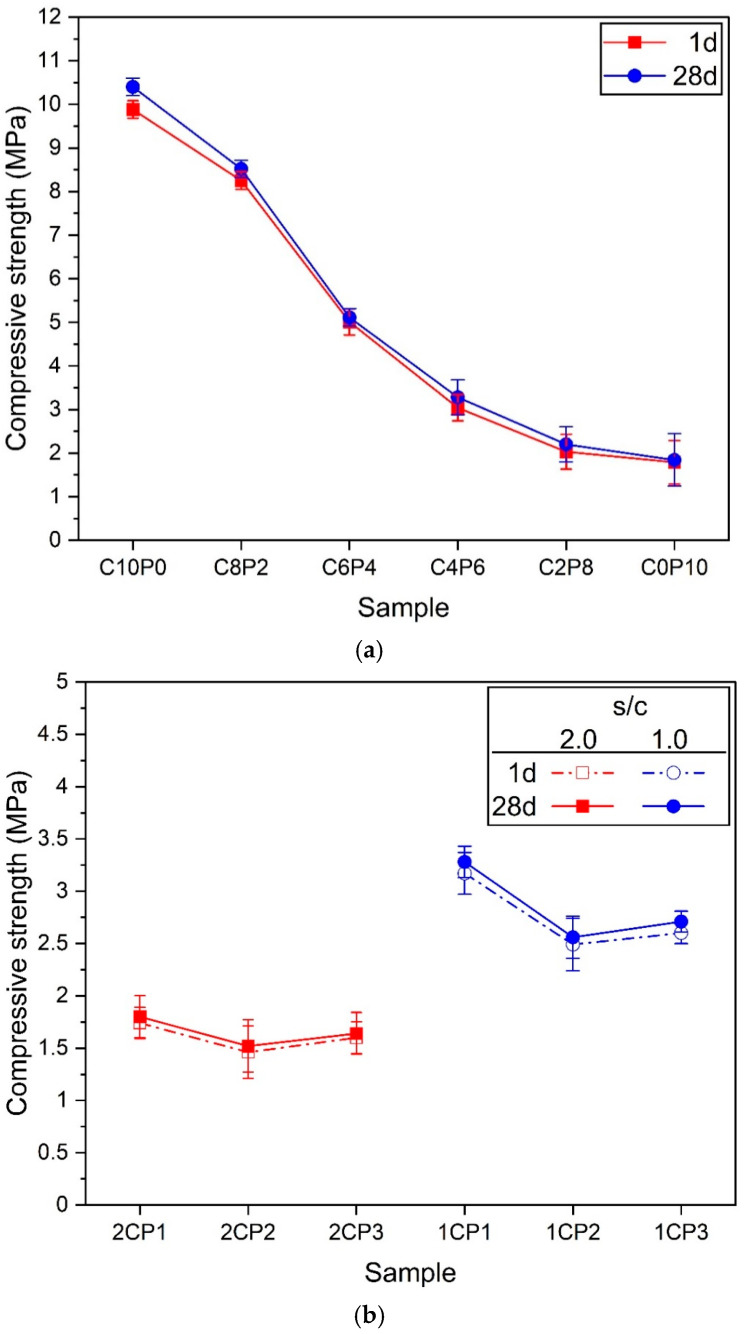
Compressive strengths of samples. (**a**) Samples according to the change in the exchange rate of CP and PL. (**b**) Samples according to the change in the amount of CP when s/c = 2.0 and 1.0.

Figure 7 shows the results of compressive strength. In Figure 7a, the compressive strength decreased steeply as the substitution ratio of PL increased. The highest compressive strength was C10P0 with 100% CP, and the lowest compressive strength was C0P10 with 100% PL. However, as the amount of PL increased, the change in strength increased due to the decrease in the mechanical performance of the sample, which led to a large deviation. Therefore, the C0P10 sample had the lowest strength and showed a large deviation at the same time. The difference in compressive strength between 1d and 28d was minimal, within 5%. There are two reasons why the difference in compressive strength values between 1d and 28d was small. First, it is thought that most of the hydration reaction of OPC particles occurred in the early-age due to the high-temperature curing applied in this experiment, and the hydration reaction in the late-age was minimal. The second is that the amount of OPC, which is relatively the heaviest (highest density), is very small to maximize the foaming effect by PVA and achieve ultralightweight. This is because the results in Figure 7 used high s/c ratios of 1.0, and 2.0.

Figure 7b shows the change in compressive strength when the amount of CP increases at two s/c ratios. Regardless of the s/c ratio, the highest compressive strength was observed when the CP:PL ratio was 1:1. And the strength decreased when it was 2:1. This is due to the formation of macropores as shown in Figure 4 and Figure 5b. Macropores form a relatively loose skeletal structure inside the matrix, which drastically reduces the mechanical performance. However, when the CP:PL ratio increased to 3:1, the compressive strength increased compared to 2:1. This is because the pores of the 3:1 samples became smaller rapidly than when it was 2:1 (Figure 4 and Figure 5b). However, the reason why the compressive strength is lower than that of the 1:1 sample is that, as explained in the dry density of Figure 6b, the pore diameter decreased rapidly, but the amount was large, so the dry density was the lowest. In addition, the s/c = 1.0 samples with a large amount of OPC showed higher compressive strength than the 2.0 samples.

The results in Figure 7 confirm that the pores formed by PVA are an important factor that reduces the mechanical performance [12,19]. And the larger the size or quantity of the pores formed inside the matrix, the lower the compressive strength [22,25,46]. The average pore diameter analysis results in Figure 5 mentioned in the previous section also showed that an increase in the PL substitution ratio increased the average pore diameter, which decreased the compressive strength as shown in Figure 7. This tendency was also observed in the samples with s/c of 2.0 and 1.0, and in particular, the strength values of the 2CP2 and 1CP2 samples with large pores were the lowest. Therefore, it is judged that sufficient consideration is needed for the formation of pores because the pores formed by PVA expand through the high-temperature curing process to improve the light weight but lower the mechanical performance.

### 3.5. Thermal Conductivity

**Figure 8:**
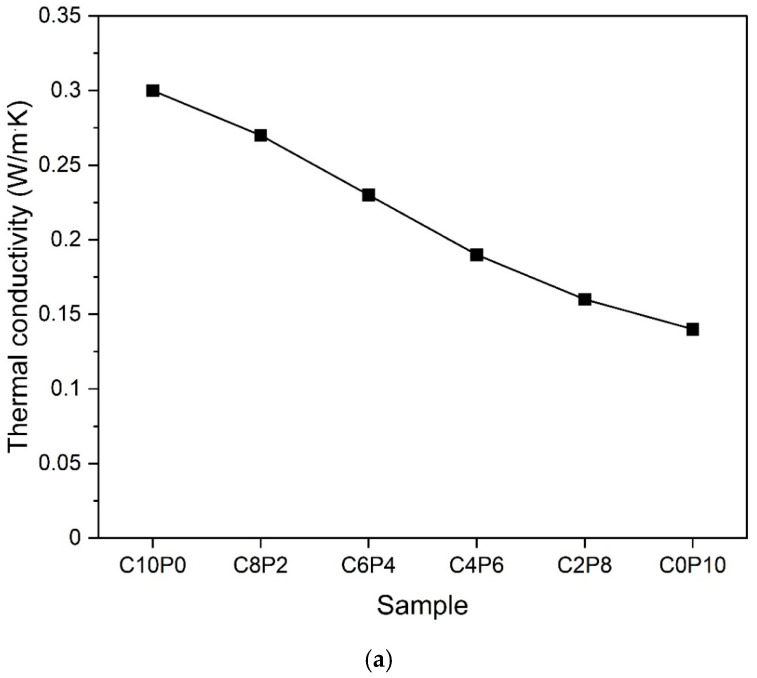

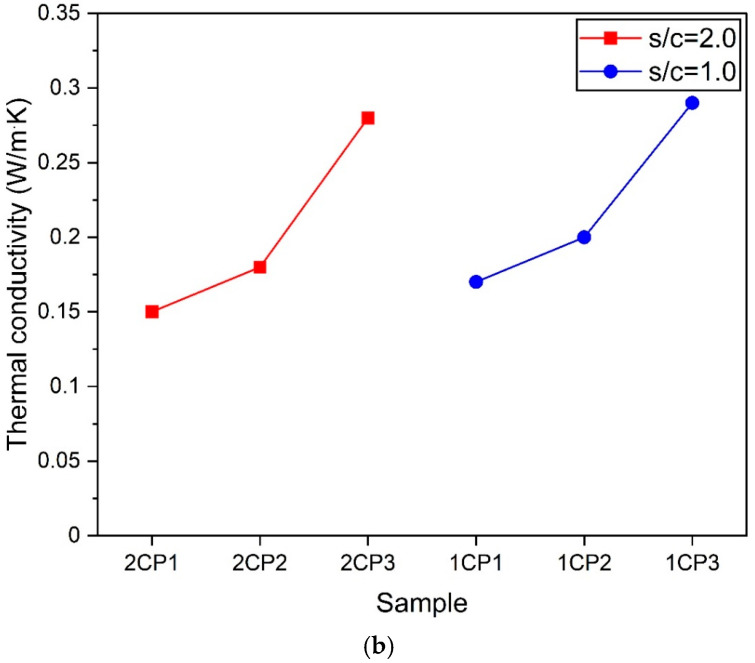
Thermal conductivity of samples. (**a**) Samples according to the change in the exchange rate of CP and PL. (**b**) Samples according to the change in the amount of CP when s/c = 2.0 and 1.0.

Figure 8 shows the measurement results for the thermal conductivity of the samples. Figure 8a shows the characteristics of the thermal conductivity according to the change in the substitution ratio of CP and PL. As the substitution ratio of PL increases, the thermal conductivity decreases, indicating that the insulation effect is improved. As a result, the C0P10 sample with 100% PL had the lowest thermal conductivity. This is due to the insulation effect of the pores distributed in the matrix as shown in Figure 3. In addition, since the PL itself is composed of very fine micropores, it has a positive effect on improving the insulation effect. Therefore, it is judged that the distribution of pores and the increase in the amount of PL have a synergistic effect of reducing the thermal conductivity. A decrease in pore diameter and pore fraction increases thermal conductivity and consequently reduces insulation performance [3]. Therefore, the size and quantity of pores created by PVA are important factors affecting insulation performance. As a result, an increase in pore diameter and quantity improves insulation performance and reduces dry density [2]. Therefore, as the substitution ratio of PL increases, the average pore diameter increases and the dry density decreases, so the insulation performance improves.

Figure 8b shows the change in the thermal conductivity according to the CP ratio when s/c is 2.0 and 1.0. When the s/c ratio is 2.0 and 1.0, the thermal conductivity of the samples with CP:PL = 1:1 (2CP1 and 1CP1) was the lowest. And as the CP:PL ratio increased from 2:1 to 3:1, the thermal conductivity value also increased, indicating a decrease in insulation performance. In Figure 4 and Figure 5b, it was confirmed that the 2:1 samples had formed large pores. These large pores rather acted as heat transfer channels and increased the thermal conductivity. In addition, the 3:1 samples showed a rapid decrease in the pore expansion effect, and the heat blocking effect was lowered due to the fine micro pores. In addition, the s/c = 1.0 samples with a large amount of OPC showed relatively high thermal conductivity compared to the 2.0 samples. This is because the average diameter of the s/c = 1.0 samples was smaller than that of the 2.0 samples in Figure 5b, and the dry density of the s/c = 1.0 samples was larger than that of the 2.0 samples in Figure 6b. In other words, the pore size of the s/c = 1.0 samples was finer than that of the 2.0 samples, and the amount of pores was relatively smaller, indicating a lower heat blocking effect [2,3].

From Figure 8, it is thought that the foaming effect of PVA and the pore expansion effect due to high-temperature curing have a positive effect on improving the insulation effect by forming pores in the matrix inside the sample. This varies depending on the size and quantity of the pores, and from Figure 5, when the average pore diameter is approximately 1.0 mm or more, it shows a low thermal conductivity of less than 0.20 W/m·K. Therefore, ultralight foam concrete using the PVA solution suggests the possibility of manufacturing a member with insulation performance.

## 4. Conclusions

The results of this experiment for the production of ultralight foam concrete using lightweight aggregates of perlite (PL) and cenosphere (CP) on an OPC base using the PVA solution are summarized as follows.

(1) It was shown that the production of ultralight foam concrete with a dry density of less than 1.0 g/cm^3^ was possible in the ranges of the PVA solution and OPC ratios (s/c) considered in this experiment, 1.0, 1.5, and 2.0, and the PVA solution–OPC–light aggregate (perlite and cenosphere) ratio (s/(c + CP + PL)) of 0.43–1.0. In addition, ultralight foam concrete with an average pore diameter of 0.1–2.3 mm and a compressive strength of 1.5–10.5 MPa could be produced.

(2) The newly proposed high-temperature curing can be suggested as an effective method for manufacturing ultralight foam concrete through the promotion of early-age hydration reaction and expansion of pores. The applied high-temperature curing method is high-temperature wet curing (60 °C, RH ≥ 90%), 12 h + high-temperature dry curing (105 ± 5 °C, RH ≤ 30%), 12 h. The high-temperature curing method can be suggested as a method to improve the problems of micropores and delayed hydration reaction, which are considered as shortcomings of existing PVA concrete.

(3) It was confirmed that the PVA solution can act as a foaming agent that exhibits a sufficient foaming effect through the high-temperature curing process. It is judged that this can be given a new role by converting the problems caused by the generation of micropores in existing concrete using PVA to a different perspective and application field. However, the expansion effect of pores is affected by the substitution ratio of CP and PL, which are lightweight aggregates. In particular, as the substitution ratio of PL increases, the expansion effect is enhanced, so the average diameter of the pores increases and the dry density decreases.

(4) It is judged that the foaming effect by the PVA solution can be applied to the manufacture of insulation materials by improving the insulation performance. In particular, the size of the foamed pores changes according to the CP–PL substitution ratio, which also affects the insulation effect. In addition, the increase in the amount of OPC showed the effects of decreasing the insulation effect, increasing the dry density, and improving the strength. Therefore, it is necessary to consider the foaming characteristics according to the selection of the amount of OPC and PL depending on the purpose.

(5) The substitution ratio of CP and PL, which are lightweight aggregates, was an important factor affecting the characteristics of ultralight foam concrete. In particular, the increase in the PL substitution ratio had a positive effect on the thermal conductivity and lightness, but it had a negative effect on the mechanical performance. In addition, at a PL substitution ratio of less than 50%, the expansion effect of the pores was minimal or pores with a large deviation of micropores and macropores were formed. Therefore, it is judged that a PL substitution ratio of 60% or more is advantageous for the formation of pores.

This study confirmed that PVA, which is different from existing foaming agents, can be used as a new foaming agent. In addition, the newly proposed high-temperature curing process showed the possibility that it can be proposed as an effective manufacturing technique for the production of ultralight foam concrete using PVA and lightweight aggregate. The ultralight foam concrete attempted in this study is expected to minimize the use of OPC and enable the production of environmentally friendly construction and building materials by using CP, PL, and PVA. In addition, it is expected to be used for the production of interior and exterior insulation materials and floating structure materials based on its lightness and insulation properties.

## Figures and Tables

**Table 1:** Chemical components of cement (OPC), perlite (PL), and cenosphere (CP).

	Chemical Composition (%)							
	SiO_2_	Al_2_O_3_	Fe_2_O_3_	MgO	CaO	K_2_O	SO_3_	Na_2_O
OPC	22.63	5.81	2.54	3.02	62.19	0.67	2.8	0.13
CP	61.07	29.46	3.17	1.24	2.06	1.59	1.01	0.17
PL	76.85	14.06	0.93	0.21	1.37	2.65	-	3.49

**Table 2:** Mix properties.

Level	PVA Dosage (%)	PVA Sol. (g)	OPC (g)	CP (g)	PL (g)	s/c	s/ (c + CP + PL)
C10P0	5	300	200	100	0	1.5	1.0
C8P2				80	20		
C6P4				60	40		
C4P6				40	60		
C2P8				20	80		
C0P10				0	100		
2CP1	5	300	150	100	100	2.0	0.85
2CP2				200	100		0.67
2CP3				300	100		0.54
1CP1			300	100	100	1.0	0.60
1CP2				200	100		0.50
1CP3				300	100		0.43

## Data Availability

Dataset available on request from the authors.
